# An Atlas of Human Anatomy

**Published:** 1879-04

**Authors:** 


*An Atlas of Human Anatomy, illustrating most of the Ordinary Dissections and many not usually practiced by the Student, accompanied by an Explanatory Text.* By RICKMAN JOHN GODLEE, M. S., F. R. C. S., Fellow of University College and Senior Demonstrator of Anatomy in the University College. Philadelphia: Lindsay and Blakiston, 1878.

We have here a most magnificent and beautifully illustrated Anatomy. All the important surgical regions are represented by colored lithographic plates, making the work when completed invaluable. It is sold in parts at $2.50 each, and should be in the hands of all physicians and surgeons. Too much cannot be said in praise of the anatomical plates. They are beautiful and true to life, as surgical guide equal to dissection, and with the explanatory text will take the place of dissection when this is impracticable. We shall have more to say of this work hereafter.

